# Impact of technology- and parent-based psychosocial interventions on family dynamics factors in children with cancer: A systematic review

**DOI:** 10.1371/journal.pone.0323483

**Published:** 2025-05-13

**Authors:** Yilin Zhang, Zitong Zhang, Yunyun Peng, Wanting Zhang, Guiyuan Ma, Sulan Lin, Carmen W.H. Chan, Ankie Tan Cheung, Jianhui Xie, Can Gu

**Affiliations:** 1 Xiangya School of Nursing, Central South University, Changsha, China; 2 Nursing School of Xinjiang Medical University, Urumqi, China; 3 The Nethersole School of Nursing, Faculty of Medicine, the Chinese University of Hong Kong, China; 4 Department of Nursing, Hunan Children’s Hospital, Changsha, China; The Chinese University of Hong Kong, HONG KONG

## Abstract

**Objective:**

This systematic review aimed to examine the impact of technology- and parent-based psychosocial interventions on family factors among children with cancer, focusing on family dynamics.

**Methods:**

Data were sourced from ten databases (CNKI, Wanfang, VIP, Sinomed, the Cochrane Library, Embase, PubMed, Web of Science, Scopus, and CINAHL) up to August 2024. The PRISMA statement guidelines, the Cochrane risk bias assessment tool, and the non-randomized controlled trial risk bias assessment tool were used in this study and experimental and quasi-experimental studies were included. The review protocol is registered in PROSPERO (CRD42023435402).

**Results:**

Twelve studies, including seven randomized controlled trials and five quasi-experimental studies, involving 1,309 parents of children with cancer, were included in the review. These studies utilized various theoretical models and delivered interventions through different modes, such as Internet-based platforms and telehealth. Overall, technology- and parent-based interventions have demonstrated positive effects on family dynamics factors, including family function, communication, coping ability, and family burden.

**Conclusions:**

Technology- and parent-based psychosocial interventions showed promise in enhancing family dynamics factors although intervention methods varied across studies. This review recommends larger-scale randomized controlled trials to evaluate the effectiveness of technology- and parent-based psychosocial interventions on family dynamics factors among this vulnerable population and highlights the potential of such interventions to improve care quality, treatment outcomes, and resource allocation in pediatric oncology.

## 1 Introduction

Childhood cancer, the sixth leading cause of cancer-related deaths, remains a major health concern [1]. An estimated 429,000 new cases of childhood cancer are reported every year worldwide [2], with an average 5-year survival rate of up to 80% for most types of cancer [3]. A cancer diagnosis profoundly impacts children and their families, leading to adverse emotional, financial, and physical outcomes [4]. As survival rates for children with cancer improve and cancer becomes a chronic condition, researchers are focusing on psychosocial issues, including mental health and quality of life [5].

Childhood cancer diagnosis and treatment can lead to numerous difficult and stressful situations for parents [6]. Indeed, parents often exhibit decreased psychological functioning, adaptation, and coping abilities [7]. Caring for a pediatric cancer patient involves navigating complex behavioral and psychosocial challenges, which can be particularly overwhelming for parents who may feel unprepared to meet their child’s needs and make informed decisions [8]. Research has indicated that robust psychosocial services are crucial for meeting the support needs of parents during and after their children’s cancer treatment [9]. As parents play a crucial role in their children’s socialization, psychological development, and overall health, their well-being is directly linked to the health outcomes of children with cancer [10]. However, much of the existing research has primarily focused on children, with less attention to the parents [11]. Therefore, evaluating the effects of interventions that support parents is essential to enhance the psychosocial well-being of both children and their parents.

The diagnosis and management of pediatric cancer can alter the whole family, leading to immediate and lasting impacts, and may cause changes in family dynamics [12]. According to Family Systems Theory, families possess inherent self-stabilizing and adaptive capacities. When faced with significant disruptions, such as a child’s cancer diagnosis, families dynamically adjust their coping strategies or restructure family roles to manage external stressors [13]. In this review, family dynamics factors refer to elements associated with parents or the entire family unit that reflect interactions within a family, such as family function, coping ability, and parent–child communication. Family function refers to the extent to which a family, as a cohesive unit, adapts to stressors, organizes daily activities, and maintains emotional and psychological stability [14]. Childhood cancer often adversely affects family function, creating conflicting dynamics and necessitating routine assessment of family function [15]. Evidence highlights the importance of improved family functions, such as enhanced cohesion, support, and expressiveness, along with reduced conflict, as these factors are linked to better adjustment in affected children [16]. However, a child’s cancer diagnosis can significantly affect the perceptions of self and family function, exposing families to the risk of adverse health outcomes [17]. Family burden, as a psychosocial stressor or structural disruption caused by critical life events such as illness, impairs family function by disrupting psychological equilibrium and reducing coping capacity [18].Therefore, researchers should focus on the role of interventions in improving family dynamics factors to mitigate the negative impact of childhood cancer.

As technology and interdisciplinary research advance, interest in technology-based psychosocial interventions has increased. Meta-analyses have shown that e-health interventions can positively impact the mental health of parents of young children, demonstrating small to moderate effects on anxiety, depression, and parenting stress [19,20–22]. The use of technology can effectively reduce accessibility barriers in children’s mental health services [23]. Technologies such as telemedicine, mobile applications, and virtual reality have now widely integrated into children and adolescents’ lives, playing a crucial role in enhancing their mental health [24,25]. In pediatric oncology, technology-driven treatments have effectively reduced negative physical and psychological symptoms in children with cancer and alleviated adverse coping and psychosocial symptoms in their parents [26].

However, to the best of our knowledge, no review has specifically examined the impact of technology- and parent-based interventions on family dynamics factors in the families of children with cancer. This systematic review sought to examine the effects of technology- and parent- based psychosocial interventions on family dynamics factors in pediatric oncology. Specifically, the review sought to (1) explore the core components and characteristics of these interventions, (2) examine the family dynamics factors they aimed to address, and (3) synthesize evidence on their reported effects and implications. This review aims to inform the development of technology-driven, family-centered care models in pediatric oncology, ultimately improving psychosocial support, care delivery, and overall family well-being.

## 2 The review

### 2.1 Design

This review follows the guidelines outlined in the Preferred Reporting Items for Systematic Reviews and Meta-Analysis statement [27] and is registered in the International Prospective Register of Systematic Reviews (CRD42023435402).

### 2.2 Search strategy

Peer-reviewed journal publications were identified by comprehensively searching 10 databases: the Chinese National Knowledge Infrastructure Database, Wanfang Database, VIP Chinese Science and Technology Periodical Database, Chinese Biomedical Literature Database, Cochrane Library, Excerpta Medica Database, PubMed, Web of Science, Scopus, and the Cumulative Index of Nursing and Allied Health Literature from establishment to August 2024. Grey literature was searched using Google and the European Grey Literature Database (www.opengrey.eu) to identify potentially omitted studies in the aforementioned search process. We also manually reviewed the reference lists of the included publications and developed search strategies for the following terms: ‘technology’, ‘telemedicine’, ‘e-health’, ‘m-Health’, ‘web-based’, ‘oncology’, ‘child’, ‘adolescent’, and ‘parents’. Medical Subject Heading (MeSH) terms and related or equivalent terms were used with search strategies adapted to the specific characteristics of each database. An example of this search strategy is presented in Table 1. For detailed information, please refer to the S1 Table.

**Table1:** Example database search, PubMed.

Main Concept	Search Formula
1.Technology	computer OR computer-based OR cyber OR cyberspace OR electronic (MeSH) OR “electronic mail” (MeSH) OR email OR e-mail OR internet (MeSH) OR internet-based OR net OR online OR virtual OR “virtual reality” OR web OR web-based OR web based OR “world wide web” OR www OR audio OR “audio-visual” OR video OR phone OR telephone (MeSH) OR “smart phone” OR “cell phone” OR “cellular phone” OR iphone OR “SMS” OR “short message service” OR “text message” OR testing OR mobile OR “mobile phone” OR ipad OR tablet (MeSH) OR “smart device” OR digital OR “personal digital assistant” OR pda OR cd-rom (MeSH) OR game OR technology (MeSH) OR technologies OR technological OR Tele-health OR telemedicine(MeSH) OR e-health OR mhealth OR Tele-Referral OR Virtual Medicine OR Tele-Intensive Care OR Mobile Health
2.Children	adolescent* OR teen* OR youth* OR child* OR infant* OR preschool OR minor*
3.Neoplasm	Neoplasm (MeSH)* OR cancer* OR tumor* OR leukemia (MeSH) OR neoplasia OR malignancy
4.Parents	parents (MeSH) OR family (MeSH) OR father (MeSH) OR mother (MeSH) OR caregivers (MeSH)
5.Study type	Clinical Trial, Randomized Controlled Trial
#1AND #2 AND #3 AND #4 AND #5	

### 2.3 Eligibility criteria

#### 2.3.1 Inclusion criteria.

1. Studies involving parents of children diagnosed with any type of cancer, aged under 18 years, were included.
2. Studies examining technology- and parent-based psychosocial interventions. Technology was defined as any type of information or communication technology, and parents were defined as at least one of the primary caregivers of children with cancer.
3. Studies with an experimental design (randomized controlled trial (RCT) design) or a quasi-experimental design (non-RCT, pre- and post-intervention design).
4. Studies using standardized outcome measures of family dynamics factors. The family dynamics factor outcomes were family communication (e.g., parent-child communication and couple communication), family coping ability (e.g., parental coping and problem-solving skills), family function (e.g., family resilience, hardiness, and adaptation), and family burden(e.g., parental care burden).

#### 2.3.2 Exclusion criteria.

1. Studies that were conference proceedings or abstracts.
2. Studies containing incomplete or suspicious data or duplicate publication.
3. Studies published in non-peer-reviewed journals.

### 2.4 Search outcome

For data management, the search results were loaded into Endnote X9, a reference management program. After discarding duplicate articles, two authors (Z. Y. L. and Z. Z. T.) independently examined and cross-checked the titles and abstracts of the remaining publications. Any dispute regarding whether to include a study was resolved following a discussion with a third author (P. Y. Y.). To extract data, the complete texts of the remaining publications were retained.

### 2.5 Data extraction

Data were independently gathered from the included publications by two authors using standardized data extraction tables, and disagreements were resolved following a discussion with a third author. The data extraction table, developed prior to the research commencement, primarily includes the following components: the year, authors, location, and design of the reported study, quantity of samples, features of the technology-based interventions used, theories used in the intervention, duration of the intervention, duration of follow-up, and significant outcomes. If data is lacking in the research, make efforts to explain and incorporate relevant findings based on the existing information in the research.

### 2.6 Quality appraisal

Two authors (Z. Y. L. and Z. Z. T.) performed independent quality assessments, and a third reviewer (P. Y. Y.) arbitrated conflict. Publications reporting RCTs were evaluated using the Cochrane Risk of Bias Tool [28], and those reporting quasi-experimental studies were evaluated using the Risk of Bias Assessment Tool for Non-Randomized Studies (ROBANS) [29]. The risk of bias was evaluated using six dimensions: selection, performance, detection, attrition, reporting, and other biases. The degree of risk of bias was evaluated and indicated as low, high, or unclear.

### 2.7 Data synthesis

We reasoned that the variety in study designs, cancer types, intervention forms, intervention durations, and measuring instruments in the studies would make a meta-analysis inappropriate. Therefore, we used narrative descriptions to summarize the included publications and relied primarily on text and tables to describe and summarize our findings. We used the general framework of narrative synthesis reported by Popay et al. [30], which included (1) a preliminary synthesis of the findings of the included studies, (2) an examination of how the interventions worked, (3) an investigation of the relationships found in the data, and (4) an evaluation of the synthesis’s robustness.

## 3 Results

### 3.1 Study selection

**Fig 1:**
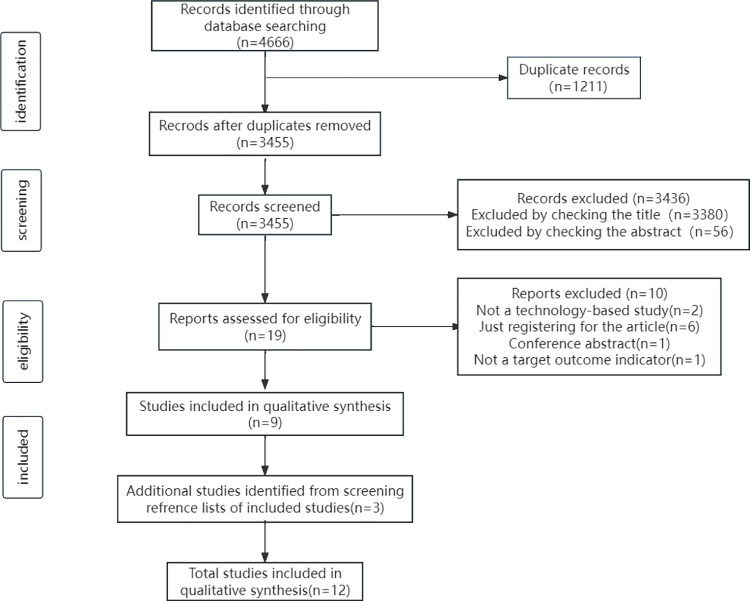
The flowchart of the review process for this study.

The database search yielded 4,666 publications, of which 1,211 were excluded as duplicates, 3,380 were excluded based on irrelevant titles, and 56 were excluded because they were abstracts without full text. From the remaining 19 full-text reviews, 2 non-technology-based intervention studies, 6 registry-only studies, 1 conference abstracts, and 1 outcome indices were excluded since they did not meet the inclusion criteria. Ultimately, nine publications (hereafter referred to as ‘studies’) were included. A review of the references of the included studies resulted in the addition of there studies that met the inclusion criteria, leading to 12 studies. A flowchart of the study selection process is illustrated in Fig 1.

### 3.2 Study characteristics

The 12 studies included 7 RCTs [31–37] and 5 quasi-experimental studies [38–42]. Of the quasi-experimental studies, four had pre- and post-designs, and one was a non-RCT. The studies were conducted in eight countries, namely, the United States, Australia, Sweden, Iceland, South Korea, Turkey, the Netherlands, and China, from 2006 to 2024. The sample included 1,309 parents of children diagnosed with various types of cancer, either in the treatment phase or during survivorship. Table 2 summarizes the authors, locations, year of publication, designs, sample sizes, characteristics of the studies, theoretical models or frameworks used in the interventions, methods of intervention delivery, instruments for measurements, and primary outcomes.

**Table 2:** Characteristics of included studies.

Author/ Country/Year	Study design	Sample characteristics	Intervention	Outcomes measurement	Results
Akard, et al. [31]/ USA/2021	● RCT ● Study duration: 3 years	● N = 97 (IC = 37, CC = 60) ● Inclusion criteria pediatric patients aged 7–17 ● Parent gender (F = 88, 93%)	● Web-based legacy intervention ● Dignity model ● Intervention duration: 2 weeks ● Comparison:usual care	● Parent-child communication ● PACS ● Baseline/post-intervention	Although there were strong intervention effects, there was no statistically significant difference in changes in parent-child communication between the groups. In contrast to standard care, legacy-making demonstrated patterns of increasing the caliber of communication—particularly father-child communication—between children and their parents over time.
Akard, et al. [32]/ USA/2021	● RCT ● Study duration: 3 years	● N = 97 (IC = 37, CC = 60) ● Inclusion criteria pediatric patients aged 7–17 ● Parent gender (F = 88, 93%)	● Web-based legacy intervention ● Dignity model ● Intervention duration: 2 weeks ● Comparison:usual care	● parental coping ● PSQ ● Baseline/post-intervention	The legacy intervention revealed trends toward parents using more primary control and disengagement coping methods over time in comparison to usual care, but there was no statistically significant difference in parental coping.
Canter, et al. [38]/ USA/2022	● One group pre-post design ● Study duration: 16 months	● N = 29 (pre = 29, post = 19) ● Inclusion criteria pediatric patients aged 0–17 ● Parent gender (F = 74%) ● Cancer type Leukemia, N = 10 (53%) Lymphoma, N = 2 (11%) Brain tumor, N = 1 (%) Solid tumor, N = 6 (32%)	● E-Health intervention with online modules and telehealth sessions ● Family system model ● Intervention duration: 3 weeks	● Family function ● The SCORE-15 ● Baseline/post-intervention	There was no statistically significant in family function, minimal effects were detected for measures of family function, warranting additional explorations (Cohen’s d = 0.15).
Canter, et al. [39]/ USA/2023	● One group pre-post design ● Study duration: 30 months	● N = 44 (pre = 44, post = 31) ● Inclusion criteria pediatric patients aged 0–17 ● Parent gender (F = 87.1%) ● Cancer type Leukemia, N = 13 (42%) Lymphoma, N = 3 (10%) Brain tumor, N = 2 (6%) Solid tumor, N = 13 (42%)	● E-Health intervention with online modules and telehealth sessions ● Family system model ● Intervention duration: 3 weeks	● Family function ● The SCORE-15 ● Baseline/post-intervention	The family functioning was noted to be statistically significant before and after the intervention, but the differences were relatively small (Cohen’s d = 0.33).
Park, et al. [35]/ South Korea/ 2023	● RCT ● Study duration: 6 months	● N = 41 (IC = 20, CC = 21) ● Inclusion criteria pediatric patients aged 0–18 ● Parent gender (F = 100%) ● Cancer type Leukemia, N = 33(80.5%) Lymphoma, N = 8 (19.5%)	● Internet-based intervention ● Walsh’s Family Resilience Framework ● Intervention duration: 4 weeks ● Comparison: usual care	● Family function ● Family resilience ● APGAR ● FRS ● Baseline/post-intervention/4 weeks post-intervention	Family function did not show any statistically significant changes between the two groups (t = 0.068, p = 0.795). When the outcome variable was compared over time in the experimental group, the family function revealed a more significant difference (β = 1.256, p = 0.018, effect size = 0.394), as did the levels of change in the overall family resilience score (β = 13.214, p = 0.003, effect size = 0.374), the belief system of the family (β = 5.288, p = 0.005, effect size = 0.382), the organization processes of the family (β = 3.932, p = 0.005, effect size = 0.394), and the communication processes of the family (β = 3.631, p = 0.018, effect size = 0.357).
Wakefield, et al. [34]/ Australia/ 2016	● RCT ● Study duration: 12 months	● N = 47 (IC = 25, CC = 22) ● Inclusion criteria pediatric patients aged 0–15 ● Parent gender (F = 72.4%) ● Cancer type CNS tumors, N = 4 (21.1%) Leukemias, N = 7 (36.8%) Lymphomas, N = 5 (26.3%) Wilms tumor, N = 3 (15.8%)	● An online cognitive behavioral therapy intervention ● The Uncertainty in illness and the Family Systems-illness models ● Intervention duration: 2 weeks ● Comparison:usual care	● Family function ● FAD ● Baseline/2 weeks post-intervention/6 months post-intervention	Group (waitlist vs. intervention) and time (baseline vs. post-intervention vs. follow-up) did not significantly affect family function.
Gårdling, et al. [40]/ Sweden/ 2018	● Two group un-matched quasi-experimental controlled clinical trial ● Study duration: 4 years	● N = 63 (IC = 32, CC = 31) ● Inclusion criteria pediatric patients aged 3–18 ● Parent gender (F = 52.4%) ● Cancer type brain tumors/solid tumors/leukemia	● Audiovisual device ● The Medical Research Council’s (MRC) framework ● Comparison: usual care	● Family function ● Parental coping ● PedsQL™ ● SOC ● First intervention/last intervention	There was no significant main effect on family function between the intervention and control groups (p = 0.269), but family function improved over time, and there were no statistically significant differences between the groups in the total and dimensional scores of parental coping (p = 0.39).
Phipps, et al. [33]/ USA/2020	● RCT ● Study duration: 50 months	● N = 621 (IC = 310, CC = 311) ● Inclusion criteria pediatric patients aged 0–18	● A web-based version delivered via mobile device ● Intervention duration: 8 weeks ● Comparison: face to face	● Problem-solving skills ● SPSI-R ● Baseline/post-intervention	The difference in problem-solving skills between the Internet-based intervention group had a statistically significant difference with a medium effect between pre- and post-intervention (effect size d = 0.32, t = 5.32, p < 0.001), and that the implementation of the intervention improved problem-solving skills in children with cancer to a certain extent, but the difference between the intervention and control groups was not statistically significant.
Ozturk, et al. [36]/ Turkey/2024	● RCT ● Study duration: 6 months	● N = 50(IC = 25, CC = 25) ● Inclusion criteria pediatric patients aged 0–18 ● Cancer type Acute lymphoblastic leukemia/ Lymphoma/Ewing sarcoma/ Osteosarcoma/Ependymoma	● M-health intervention with video instructions ● Intervention duration: 8 weeks ● Comparison:usual care	● Stress coping ● SCS ● Baseline/post-intervention	At baseline, mean SCS scores were similar between the intervention (59.24 ± 21.26) and control (60.28 ± 2.08) groups. After the intervention, the intervention group’s mean SCS scores rose to 91.04 ± 1.57, significantly higher than the control group (58.16 ± 2.55, p < 0.05). Subdimension scores—problem avoidance (31.16 ± 0.59), problem orientation (30.84 ± 0.88), and social support seeking (29.04 ± 0.75)—were also significantly higher in the intervention group compared to the control group (19.44 ± 1.17; 18.80 ± 0.92; 19.72 ± 0.96). The intervention group showed significant reductions in SCS scores post-program, while no significant changes occurred in the control group (p > 0.05).
Joosten, et al.[37]/ Netherlands/2024	● RCT ● Study duration: 16 months	● N = 100(IC = 50, CC = 50) ● Inclusion criteria pediatric patients aged 0–18 ● Cancer type Hematology/Solid tumors/Neuro	● Op Koers intervention with online chat sessions and a booster session ● Intervention duration: 6 weeks ● Comparison:usual care	● Coping skills ● DCRQ ● Baseline/6 weeks post-intervention/6 months post-intervention/12 months post-intervention	The intervention significantly improved the use of relaxation coping skills at T1 (β = 0.35) and T2 (β = 0.32). However, no significant effects were observed for open communication, positive thinking, or predictive control coping skills. In the intervention group, relaxation continued to improve from T2 to T3 (β = 0.28).
Wang, et al. [41]/China/ 2018	● Two group ● quasi-experimental pre-post design ● Study duration: 7 months	● N = 101 (IC = 51, CC = 50) ● Inclusion criteria pediatric patients aged 0–15 ● Cancer type Acute lymphoblastic leukemia	● MHealth supportive care intervention with a smartphone App and WeChat Official Account ● The Supportive Care Needs Framework ● Intervention duration: 3 months ● Comparison:usual care	● Burden of Care ● ZBI ● Baseline/post-intervention	The findings demonstrated that parents’ care load grew significantly (P = 0.01) during the course of the three-month observation period. While there was no significant rise in the intervention group and no significant difference between the two groups (P = 0.62, P = 0.08), the care load of the parents in the observation group increased significantly (P = 0.09).
Svavarsdottir,et al. [42]/ Iceland/ 2006	● One group ● Pre-post design ● Study duration: 26 months	● N = 19 (pre = 19, post = 18) ● Inclusion criteria pediatric patients aged 0–18 ● Parent gender (F = 50%) ● Cancer type lymphoma, N = 1 (10%) leukemia, N = 5 (50%) brain tumors, N = 2 (20%) Sarcoma, N = 2 (20%)	● Internet support ● interviews ● The Calgary Family Intervention Model (CFIM) ● Intervention duration: 6 months	● Parental coping ● Family strength and resources ● Family adaptation ● CHIP ● FHI ● FAS ● Baseline/post-intervention/one-year post-intervention	There were no significant differences were founded among parental coping, parents’ family hardiness and adaptation before and after the intervention. But it was found that there is a trend towards increased use of coping strategies by parents, and mothers’ perceptions of both the family hardiness and adaptation decreased, and fathers’ perceptions of their family hardiness remained unchanged, and the family adaptation decreased slightly over time.

Abbreviations: RCT: randomized controlled trial; IC: intervention group; CC: control group; F: female; PACS: the Parent Adolescent Communication Scale; PSQ: the Response to Stress Questionnaire; The SCORE-15: the 15-item Portuguese version of the Systemic Clinical Outcome Routine Evaluation; APGAR: the Adaptation, Partnership, Growth, Affection, and Resolve scale; FRS: Family Resiliency Scale; FAD: the McMaster Family Assessment Device; PedsQL™: the Swedish version of the questionnaire PedsQL™Family Impact Module; SOC: the Swedish version of the Sense of Coherence questionnaire; SPSI-R: the Social Problem-Solving Inventory, Revised; SCS: the Stress Coping Scale; DCRQ: Disease-related coping skills questionnaire; ZBI: the Zarit Burden Inventory; CHIP: the Coping Health Inventory for Parents; FHI: the Family Hardiness Index; FAS: the Family Adaptation Scale;

### 3.3 Risk of bias

**Fig 2:**
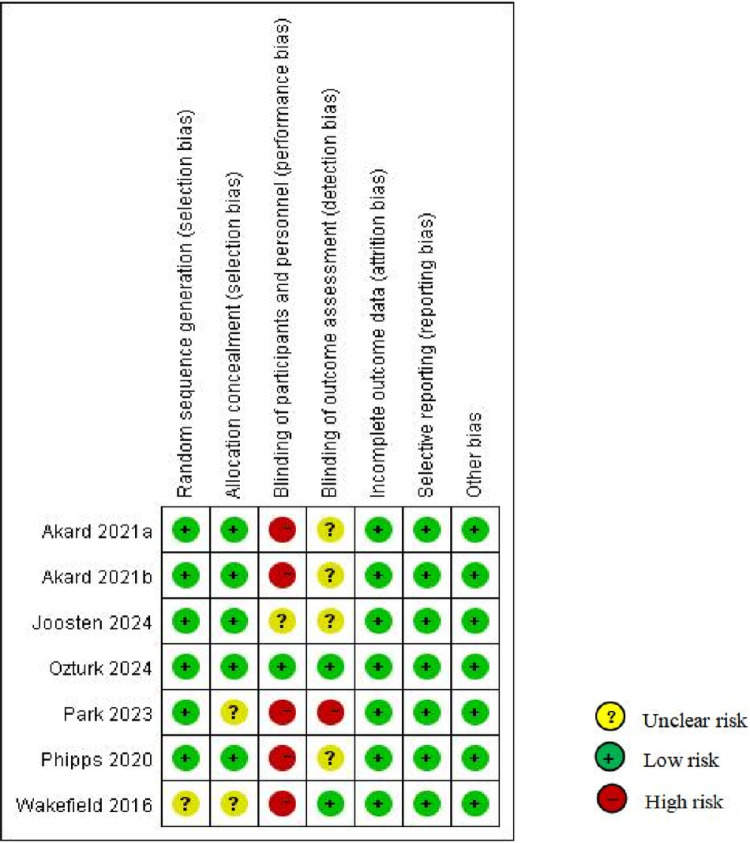
Randomized experimental studies.

**Fig 3:**
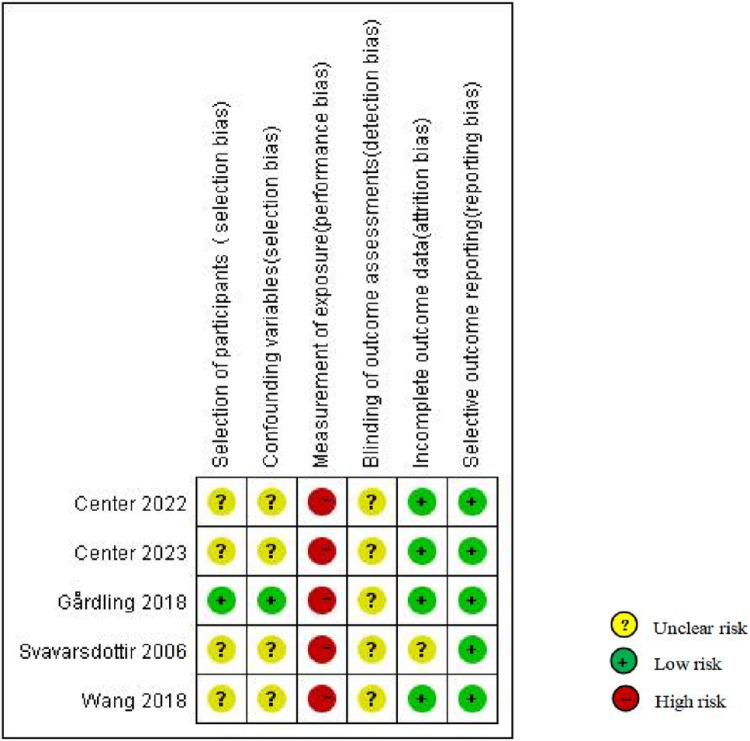
Quasi-experimental studies.

Fig 2 and Fig 3 illustrates the risk of bias in the included studies. Using the Cochrane Risk of Bias tool, six RCTs employed random sequence generation and networked computers for random allocation, resulting in a low risk of bias in terms of allocation concealment [31–33,35–37]. Two studies reported blinded outcome assessments [34,36], whereas another reported that the outcome assessments were not blinded and that the intervention was provided and evaluated by the same researchers [35]. Consequently, these seven studies exhibited a high risk of performance bias but a low risk of attrition, reporting, and other biases.

Quasi-experimental studies revealed various risk levels. One was a non-randomized controlled study with a clearly described participant selection, thus having a low risk of bias [40]. The remaining four quasi-experimental studies that employed pre- and post-control designs did not describe the participant selection [38,39,41,42]. One study accounted for confounding variables and noted no significant differences in background variables across groups [40]. All five studies delivered standardized interventions; however, their primary outcomes were measured using self-report methods, which might have influenced the results. None of the studies described the blinding of outcome assessments. One study did not mention the treatment of incomplete data [42], whereas the remaining four did. These studies generally have a low risk of bias in selective outcome reporting.

### 3.4 Results of individual studies

#### 3.4.1 Theoretical models used in the interventions.

Of the 12 studies, 11 were based on specific theoretical models or frameworks. Specifically, two studies used the dignity model [31,32], two used the family systems theory [38,39], and the remaining were based on various theoretical foundations, including Walsh’s family resilience framework [35], the Uncertainty in illness and the Family Systems-illness models [34], the Medical Research Council’s (MRC) Framework [40], the Supportive Care Needs Framework [41], the Calgary Family Intervention Model (CFIM) [42], the Smith’s Attentional Behavioral Cognitive (ABC) relaxation theory [36], the disability-stress-coping model [37].

#### 3.4.2 Mode of delivery.

Interventions in the 12 included studies varied in form and content: Five used Internet-based interventions, such as online manuals and videos, to improve family function and family resilience [35], family hardiness and adaptation [42], parental coping [32,33,42], and the quality of parent-child communication [31]. Two studies conducted legacy interventions based on telehealth sessions to explore their role in improving family function [38,39]. Two studies provided online courses with mobile software devices to explore the role of interventions in improving family function [34], reducing parental caregiving burden [41] and improving parental coping [37]. Two studies used audiovisual devices to provide parents with audiobooks or videos to improve their ability to cope with life stressors and family function [36,42].

#### 3.4.3 Family dynamics factors outcome.

The included studies demonstrated marked differences in terms of result validity. Outcome indicators focused on improving family communication, family function, coping ability, and reducing family burden. One study [31] found no significant difference in parent–child communication between the intervention and control groups (P > 0.05, Cohen’s d ranged from -0.20 to 0.33), although family communication was an improvement, particularly between fathers and children(Cohen’s d = −0.22 to 0.33). Six studies reported improvements in family function after the intervention. However, the results were inconsistent; some studies found no significant effects, whereas others documented significant improvements in family function post-intervention. Two studies found no significant differences in family function between groups [34,42]. Wakefield et al. [34] found no significant effect of time on family function at baseline, post-intervention, or follow-up. However, Park et al. [35] noted significant improvements in family function pre-intervention, immediately post-intervention, and at four weeks post-intervention, with a medium effect size and within-group changes over time in the intervention group (β= 1.256, p = 0.018, effect size = 0.394). Center et al. [38,39] reported small significant changes in family function post-intervention compared with pre-intervention (95% CI = -0.14,0.79; Cohen’s d = 0.33). Svavarsdottir et al. [42] found no significant differences in family hardiness and adaptation before and after intervention (P > 0.05). One study [40] found no significant between-group differences in coping abilities (P = 0.39). However, similar to two other studies, there was an increasing trend toward using coping strategies over time [32,42]. One RCT found a significant difference and medium effect size in parental coping pre- and post-intervention in an Internet-based group (effect size = 0.32, t = 5.32, p < 0.001), although the difference between the intervention and control groups was not significant (P = 0.55) [33]. Two RCTs found significant improvements in parental coping skills in the intervention group compared with the control group (P ＜ 0.05) [36,37]. One study [41] found no significant difference in caregiving burden between intervention and control groups (P = 0.86). However, the burden significantly increased in the control group but not the intervention group, likely due to additional support. In summary, the technology- and parent-based interventions demonstrate beneficial effects on family dynamics factors including improved family function, enhanced parent-child communication, strengthened coping ability, and reduced family burden.

## 4 Discussion

This review systematically analyzed 12 studies to elucidate the implementation and characteristics of current technology- and parent-based psychosocial interventions for children with cancer and report the family dynamics factors measured in these studies and the impact of interventions on these family dynamics factors. Our findings suggest that technology- and parent-based interventions positively affect family dynamics factors particularly family function, communication, coping ability, and burden. Six studies reported benefits in family function [34,35,38–40,42], with two showing significant improvements pre- and post-intervention [35,39]. One study found beneficial improvements in parent-child communication, particularly between fathers and their children, following both technology- and parent-based interventions [31]. Six studies reported improvements in family coping abilities after technology- and parent-based interventions [32,33,36,37,40,42]. One study found no significant increase in caregiver burden after technology - and parent-based interventions [41].

### 4.1 Evaluation of bias and effectiveness in the included studies

Overall, RCTs had a relatively low risk of bias based on quality assessment tools. In contrast, the quality of the quasi-experimental studies varied slightly, primarily due to differences in selection and attrition bias. All studies had a high risk of selection bias since psychosocial interventions often expose participants to the intervention environment, making it impossible to blind them to the intervention. Among the seven RCTs, six reported a low risk of selection bias [31–33,35–37]. RCTs evaluating the effectiveness of medical interventions on health outcomes produced the fewest outcome biases [43]. Large-sample RCTs have demonstrated good effectiveness [33], indicating that large-scale RCTs should be conducted to explore the effectiveness of technology- and parent-based psychosocial interventions in relation to family dynamics factors. The remaining five studies were quasi-experimental, with relatively small sample sizes (n = 19–63) and single-center designs, which necessitated detailed consideration of the implausibility of the experimental results owing to the selection bias, showing a low risk [40]. One quasi-experimental study was conducted in China [41], whereas the remaining in developed countries. Therefore, researchers should consider conducting additional studies on technology- and parent-based interventions tailored to populations in developing countries. Overall, we recommend conducting further large-sample, multicenter RCTs to produce more generalizable and transferable findings than those of previous RCTs.

### 4.2 Forms of technology- and parent-based psychosocial interventions

This review revealed significant variations in the form and duration of technology- and parent-based psychosocial interventions for children with cancer. Despite this variability, all included studies reported at least one positive effect of family dynamics factors.

Currently, there is no clear prescriptive theoretical framework for the form and duration of these interventions. Although standardized programs can enhance their application and promotion, the diverse and unstructured nature of technology makes it challenging to develop systematic intervention programs. In the reviewed studies, the intervention duration ranged from two weeks to six months. A similar review of e-Health and m-Health interventions showed a wide range of durations, highlighting the need to further investigate these factors in pediatric oncology [44]. A systematic review and meta-analysis of technology-based parenting interventions for children’s physical and psychological health found that the duration of the interventions ranged from two weeks to 4 months [45]. These findings indicate the importance of exploring the duration of interventions in pediatric oncology.

Interventions utilizing the internet, mobile devices, and telemedicine are user-friendly, scalable, and customizable. These features are particularly advantageous for families living in remote areas, potentially reducing travel costs and making healthcare more accessible. A systematic economic evaluation is required to assess the cost-effectiveness of technology- and parent-based psychosocial interventions. This would help clarify the potential cost savings and their impact on the psychosocial well-being of families with children with cancer.

### 4.3 Effectiveness of technology- and parent-based psychosocial intervention on family dynamics factors

This systematic review found that technology- and parent-based interventions could improve family dynamics factors particularly by enhancing family communication, family function, coping ability, and reducing family burden. A similar review identified a link between effective parent and child communication and positive mental health outcomes during childhood cancer treatment [46]. These findings support the role of technology- and parent-based interventions as crucial components of comprehensive care for children with cancer. However, unlike other studies, one indicated that e-health interventions for parents did not enhance family function [47]. Although clinical efficacy was not consistently confirmed across all the included studies, this finding provides a foundation for further research. Notably, the high variability in different interventions and settings may stem from differences in healthcare systems across various countries.

From a healthcare research perspective, more high-quality studies are required to investigate the efficacy of technology- and parent-based psychosocial interventions for family dynamics factorsin childhood cancers. Therefore, conducting more rigorous RCTs is essential. Future research should address poor reporting quality by adhering to established reporting standards. Overall, the interventions included in this review improved family dynamics factors in children with cancer. To the best of our knowledge, this is the first review to address the impact of technology- and parent-based psychosocial interventions on family dynamics factors in families of children with cancer.

## 5 Limitation

This study has some limitations. First, given the small sample sizes of the included studies and the varying quality of the tools used to evaluate the outcome indicators, no meta-analysis was conducted to quantify the effect sizes of the interventions. Second, five studies were pilot studies that explored the feasibility and acceptability of technology- and parent-based interventions and reported only preliminary results.

## 6 Implication of practice

This systematic review highlights several key implications for the practice of technology- and parent-based psychosocial interventions in families of children with cancer. The findings suggest that these interventions can significantly enhance family dynamics factors such as communication, function, and coping ability and reduce family burden. Therefore, healthcare professionals should consider incorporating these interventions into standard care practices to bolster family support systems—for instance, by designating intervention coordinators in pediatric units to facilitate family referral and program monitoring, ensuring seamless integration of technology or in-person support. Given the variability in the form and duration of these interventions, personalized approaches are crucial. Clinicians should conduct thorough family assessments to tailor interventions, such as adapting communication modules for culturally diverse family structures (e.g., multigenerational caregiving), optimizing technological tools for accessibility, or adjusting intervention intensity to align with treatment phases. This will help to create more universally applicable guidelines and improve the global care standards for pediatric oncology.

## 7 Conclusion

The technology- and parent-based psychosocial interventions examined in this review demonstrated efficacy in enhancing family dynamics factors in the families of children with cancer. After the intervention, family dynamics factors such as family communication, family function, family coping ability, and family burden, improved. Technology- and parent-based psychosocial interventions are crucial components of comprehensive care for children with cancer. Future research should focus on rigorous multicenter RCTs and adhere to established reporting standards to strengthen the evidence base and enhance the applicability of these interventions.

## Supporting information

S1 Table Search strategy. (DOCX)

S2 Table List of all the extracted studies for secondary screening. (DOCX)

S1 File Data extraction form. (XLSX)

S2 File PRISMA_2020_checklist. (DOCX)

S3 File PROSPERO registration. (PDF)

S4 File Quality evaluation information form. (XLSX)
