# Supplementary material for: Impact of technology- and parent-based psychosocial interventions on family dynamics factors in children with cancer: A systematic review
Source: PLoS One. 2025 May 13;20(5):e0323483. doi: 10.1371/journal.pone.0323483 (PMC12074529; doi:10.1371/journal.pone.0323483)
Supplement: S1 Table — (DOCX) [file pone.0323483.s001.docx]

**Search Strategies**

| **Database** | **Final search date** | **Search Strategies** | **Result** |
| --- | --- | --- | --- |
| **PubMed** | **01/08/2023** | **(((computer OR computer-based OR cyber OR cyberspace OR electronic OR "electronic mail" OR email OR e-mail OR internet OR internet-based OR net OR online OR virtual OR "virtual reality" OR web OR web-based OR web based OR "world wide web" OR www OR audio OR "audio-visual" OR video OR phone OR telephone OR "smart phone" OR "cell phone" OR "cellular phone" OR iphone OR "SMS" OR "short message service" OR "text message" OR testing OR mobile OR "mobile phone" OR ipad OR tablet OR "smart device" OR digital OR "personal digital assistant" OR pda OR cd-rom OR game OR technology OR technologies OR technological OR Tele-health OR telemedicine OR e-health OR mhealth OR Tele-Referral OR Virtual Medicine OR Tele-Intensive Care OR Mobile Health) AND (adolescent* OR teen* OR youth* OR child* OR infant* OR preschool OR minor*)) AND (neoplasm* OR cancer* OR tumor* OR leukemia OR neoplasia OR malignancy)) AND (****parent OR family OR father OR mother OR caregivers) Filters: Clinical Trial, Randomized Controlled Trial** | **1190** |
| **Web of Science** | **01/08/2023** | #1 TS=(computer OR computer-based OR cyber OR cyberspace OR electronic OR "electronic mail" OR email OR e-mail OR internet OR internet-based OR net OR online OR virtual OR "virtual reality" OR web OR web-based OR web based OR "world wide web" OR www OR audio OR "audio-visual" OR video OR phone OR telephone OR "smart phone" OR "cell phone" OR "cellular phone" OR iphone OR "SMS" OR "short message service" OR "text message" OR testing OR mobile OR "mobile phone" OR ipad OR tablet OR "smart device" OR digital OR "personal digital assistant" OR pda OR cd-rom OR game OR technology OR technologies OR technological OR Tele-health OR telemedicine OR e-health OR mealth OR Tele-Referral OR Virtual Medicine OR Tele-Intensive Care OR Mobile Health)  #2 TS=(adolescent* OR teen* OR youth* OR child* OR infant* OR preschool OR minor*)  #3TS=(neoplasm* OR cancer* OR tumor* OR leukemia OR neoplasia OR malignancy)  #4 TS=(parent OR family OR father OR mother OR caregivers)  #1 and #2 and #3 and #4 and Clinical Trial (文献类型) | **1219** |
| **Embase** | **02/08/2023** | ti,ab,kw:(computer OR 'computer based' OR cyber OR cyberspace OR electronic OR 'electronic mail' OR email OR 'e mail' OR internet OR 'internet based' OR net OR online OR virtual OR 'virtual reality' OR web OR 'web based' OR 'world wide web' OR www OR audio OR 'audio-visual' OR video OR phone OR telephone OR 'smart phone' OR 'cell phone' OR 'cellular phone' OR iphone OR 'sms' OR 'short message service' OR 'text message' OR testing OR mobile OR 'mobile phone OR ipad OR tablet OR 'smart device' OR digital OR 'personal digital assistant' OR pda OR 'cd rom' OR game OR technology OR technologies OR technological OR 'tele health' OR telemedicine OR 'e health' OR mhealth OR 'tele referral' OR 'virtual medicine' OR 'tele-intensive care OR 'mobile health' ) AND (adolescent* OR teen* OR youth* OR child* OR infant* OR preschool OR minor* ) AND (neoplasm* OR cancer* OR tumor* OR leukemia OR neoplasia OR malignancy) AND (parent OR family OR father OR mother OR caregivers )  AND ('clinical trial'/de OR 'controlled study'/de OR 'randomized controlled trial'/de)  AND 'article'/it | **218** |
| **The cochrane library trials** | **01/08/2023** | computer OR computer-based OR cyber OR cyberspace OR electronic OR "electronic mail" OR email OR e-mail OR internet OR internet-based OR net OR online OR virtual OR "virtual reality" OR web OR web-based OR web based OR "world wide web" OR www OR audio OR "audio-visual" OR video OR phone OR telephone OR "smart phone" OR "cell phone" OR "cellular phone" OR iphone OR "SMS" OR "short message service" OR "text message" OR testing OR mobile OR "mobile phone" OR ipad OR tablet OR "smart device" OR digital OR "personal digital assistant" OR pda OR cd-rom OR game OR technology OR technologies OR technological OR Tele-health OR telemedicine OR e-health OR mhealth OR Tele-Referral OR Virtual Medicine OR Tele-Intensive Care OR Mobile Health in Title Abstract Keyword AND adolescent* OR teen* OR youth* OR child* OR infant* OR preschool OR minor* in Title Abstract Keyword AND neoplasm* OR cancer* OR tumor* OR leukemia OR neoplasia OR malignancy in Title Abstract Keyword AND parent OR family OR father OR mother OR caregivers in Title Abstract Keyword - (Word variations have been searched) | **1563** |
| **Scopus** | **01/08/2023** | #1 TITLE-ABS-KEY (computer OR computer-based OR cyber OR cyberspace OR electronic OR "electronic mail" OR email OR e-mail OR internet OR internet-based OR net OR online OR virtual OR "virtual reality" OR web OR web-based OR web based OR "world wide web" OR www OR audio OR "audio-visual" OR video OR phone OR telephone OR "smart phone" OR "cell phone" OR "cellular phone" OR iphone OR "SMS" OR "short message service" OR "text message" OR testing OR mobile OR "mobile phone" OR ipad OR tablet OR "smart device" OR digital OR "personal digital assistant" OR pda OR cd-rom OR game OR technology OR technologies OR technological OR Tele-health OR telemedicine OR e-health OR mhealth OR Tele-Referral OR Virtual Medicine OR Tele-Intensive Care OR Mobile Health )  #2TITLE-ABS-KEY( adolescent* OR teen* OR youth* OR child* OR infant* OR preschool OR minor* )  #3TITLE-ABS-KEY( neoplasm* OR cancer* OR tumor* OR leukemia OR neoplasia OR malignancy )  #4 TITLE-ABS-KEY (parent OR family OR father OR mother OR caregivers )  #1 AND #2 AND #3 AND #4 AND ( LIMIT-TO ( DOCTYPE , "ar" ) | **85** |
| **CINACL** | **01/08/2023** | computer OR computer-based OR cyber OR cyberspace OR electronic OR "electronic mail" OR email OR e-mail OR internet OR internet-based OR net OR online OR virtual OR "virtual reality" OR web OR web-based OR web based OR "world wide web" OR www OR audio OR "audio-visual" OR video OR phone OR telephone OR "smart phone" OR "cell phone" OR "cellular phone" OR iphone OR "SMS" OR "short message service" OR "text message" OR testing OR mobile OR "mobile phone" OR ipad OR tablet OR "smart device" OR digital OR "personal digital assistant" OR pda OR cd-rom OR game OR technology OR technologies OR technological OR Tele-health OR telemedicine OR e-health OR mhealth OR Tele-Referral OR Virtual Medicine OR Tele-Intensive Care OR Mobile Health ) AND ( adolescent* OR teen* OR youth* OR child* OR infant* OR preschool OR minor* ) AND ( neoplasm* OR cancer* OR tumor* OR leukemia OR neoplasia OR malignancy ) AND ( parent OR family OR father OR mother OR caregivers )and Clinical Trial | **37** |
| **CNKI** | **01/08/2023** | #1主题：互联网 + 在线 + 远程医学 + 远程医疗 + 移动医疗 + 虚拟现实 + 视听设备 + 多媒体 + 移动应用 + APP + 电信 + 电话 + 视频会议 + 电子邮件 + 短信 + 手机 + 微信 + QQ + 软件 + 游戏  #2 主题：肿瘤 + 癌症 + 瘤 + 白血病  #3主题：儿童 + 学龄前儿童 + 青少年 + 婴幼儿 + 未成年人  #1 AND #2 AND#3AND期刊论文 | **110** |
| **Wangfang** | **01/08/2023** | #1互联网 OR 在线 OR 远程医学 OR 远程医疗 OR 移动医疗 OR 虚拟现实 OR 视听设备 OR 多媒体 OR 移动应用 OR APP OR 电信 OR 电话 OR 视频会议 OR 电子邮件 OR 短信 OR 手机 OR 微信 OR QQ OR 软件 OR 游戏  #2 肿瘤 OR 癌症 OR 瘤 OR 白血病  #3儿童 OR 学龄前儿童 OR 青少年 OR 婴幼儿 OR 未成年人  #4家庭 OR 父母 OR 父亲 OR 母亲 OR 双亲 OR 照护者  #1 AND #2 AND #3 AND #4 | **106** |
| **VIP** | **01/08/2023** | [#1 题名或关键词=互联网 OR 在线OR 远程医学 OR 远程医疗OR 移动医疗 OR 虚拟现实 OR 视听设备OR 多媒体OR 移动应用OR APP OR 电信OR电话 OR 视频会议OR 电子邮件 OR 短信OR手机OR 微信OR QQ OR软件OR游戏](http://qikan.cqvip.com/Qikan/search/index?LngMySearHistoryIdGuid=c238f29f-e8f7-444d-926c-fc217ff65f60&from=Qikan_Article_History)  [#2 题名或关键词=肿瘤 OR 癌症OR 瘤 OR 白血病](http://qikan.cqvip.com/Qikan/search/index?LngMySearHistoryIdGuid=c238f29f-e8f7-444d-926c-fc217ff65f60&from=Qikan_Article_History)  [#3题名或关键词=儿童 OR 学龄前儿童 OR 青少年 OR 婴幼儿 OR 未成年人](http://qikan.cqvip.com/Qikan/search/index?LngMySearHistoryIdGuid=c238f29f-e8f7-444d-926c-fc217ff65f60&from=Qikan_Article_History)  #1 AND #2 AND #3 | **128** |
| **SinoMed** | **01/08/2023** | [#1 "互联网" OR "在线" OR "远程医学" OR "远程医疗" OR "移动医疗" OR "虚拟现实" OR "视听设备" OR "多媒体" OR "移动应用" OR "APP" OR "电信" OR "电话" OR "视频会议" OR "电子邮件" OR "短信" OR "手机" OR "微信" OR "QQ" OR "软件" OR "游戏"](javascript:toDoRelimitSearch();)  [#2 "肿瘤" OR "癌症" OR "瘤" OR "白血病"](javascript:toDoRelimitSearch();)  [#3 "儿童" OR "学龄前儿童" OR "青少年" OR "婴幼儿" OR "未成年人"](javascript:toDoRelimitSearch();)  [#1 AND #2 AND #3 AND ("临床试验"[文献类型] OR "随机对照试验"[文献类型] OR "多中心研究"[文献类型])](javascript:toDoRelimitSearch();) | **10** |
