# Supplementary material for: Impact of technology- and parent-based psychosocial interventions on family dynamics factors in children with cancer: A systematic review
Source: PLoS One. 2025 May 13;20(5):e0323483. doi: 10.1371/journal.pone.0323483 (PMC12074529; doi:10.1371/journal.pone.0323483)
Supplement: S2 Table — (DOCX) [file pone.0323483.s002.docx]

**Supplement Table2: List of all the extracted studies for secondary screening (N=22)**

| **Number** | **Reference** | **Included/ Excluded Decision** | **Reasons for exclusion (if excluded)** |
| --- | --- | --- | --- |
| 1 | Canter KS, Vega G, Perez Ramirez A, Munoz Osorio A, Thomas C, Lewis AM, et al. Acceptability and Feasibility of eSCCIP: Results From a Pilot Study of the Electronic Surviving Cancer Competently Intervention Program. J Pediatr Psychol. 2023;48(3):216-27.Doi: 10.1093/jpepsy/jsac082. | Included | NA |
| 2 | Canter KS, McIntyre R, Babb R, Ramirez AP, Vega G, Lewis A, et al. A community-based trial of a psychosocial eHealth intervention for parents of children with cancer. Pediatr Blood Cancer. 2022;69(1):e29352.Doi: 10.1002/pbc.29352. | Included | NA |
| 3 | Wakefield C, Sansom-Daly U, Brittany M, Robertson E, Ellis S, Marshall K, et al. 'Cascade': a randomized trial of online support for parents after their child's cancer treatment. 2016;25:106-7.Doi: 10.1002/pon.4272. | Included | NA |
| 4 | Park M, Kim S, Lee H, Shin YJ, Lyu CJ, Choi EK. Development and effects of an internet-based family resilience-promoting program for parents of children with cancer: A randomized controlled trial. Eur J Oncol Nurs. 2023;64.Doi: 10.1016/j.ejon.2023.102332. | Included | NA |
| 5 | Akard TF, Dietrich MS, Friedman DL, Wray S, Gerhardt CA, Given B, et al. Effects of a Web-Based Pediatric Oncology Legacy Intervention on Parental Coping. 2021;48(3):309-16.Doi: 10.1188/21.ONF.309-316. | Included | NA |
| 6 | Gårdling J, Törnqvist E, Månsson ME, Hallström IK. Impact of Age-appropriate Preparations for Children With Cancer Undergoing Radiotherapy on Parents and Family Functioning, Parents' Anxiety and Hospital Costs - A Feasibility Study. J Pediatr Nurs. 2018;43:e51-8.Doi: 10.1016/j.pedn.2018.09.004. | Included | NA |
| 7 | Akard TF, Dietrich MS, Friedman DL, Gerhardt CA, Given B, Hendricks-Ferguson V, et al. Improved parent–child communication following a RCT evaluating a legacy intervention for children with advanced cancer. Prog Palliat Care. 2021;29(3):130-9.Doi: 10.1080/09699260.2020.1826778. | Included | NA |
| 8 | Phipps S, Fairclough DL, Noll RB, Devine KA, Dolgin MJ, Schepers SA, et al. In-person vs. web-based administration of a problem-solving skills intervention for parents of children with cancer: report of a randomized noninferiority trial. 2020;24:100428.Doi: 10.1016/j.eclinm.2020.100428. | Included | NA |
| 9 | Ozturk CS, Katikol E. Effect of mHealth-based relaxation program on stress coping and anxiety levels in mothers of children with cancer: A randomized controlled study. Patient Educ Couns. 2024;123.Doi: 10.1016/j.pec.2024.108247. | Included | NA |
| 10 | Wang J, Howell D, Shen N, Geng Z, Wu F, Shen M, et al. mHealth Supportive Care Intervention for Parents of Children With Acute Lymphoblastic Leukemia: Quasi-Experimental Pre- and Postdesign Study. Jmir Mhealth Uhealth. 2018;6(11):e195.Doi: 10.2196/mhealth.9981. | Included | NA |
| 11 | Joosten M, Maurice-Stam H, van Gorp M, Beek LR, Stremler-van HD, Scholten L, et al. Efficacy of Op Koers Online, an online group intervention for parents of children with cancer: Results of a randomized controlled trial. Psycho-Oncology. 2024;33(1):e6284.Doi: 10.1002/pon.6284. | Included | NA |
| 12 | Svavarsdottir EK, Sigurdardottir AO. Developing a family-level intervention for families of children with cancer. Oncol Nurs Forum. 2006;33(5):983-90.Doi: 10.1188/06.ONF.983-990. | Included | NA |
| 13 | NCT. A Web-Based Stem Cell Transplant Support System or Standard Care in Young Patients Undergoing Stem Cell Transplant and Their Families. 2008. | Excluded | Just registering for the article |
| 14 | Cho E, Dietrich MS, Friedman DL, Gilmer MJ, Gerhardt CA, Given BA, et al. Effects of a Web-Based Pediatric Oncology Legacy Intervention on the Coping of Children With Cancer. Am J Hosp Palliat Me. 2023;40(1):34-42.Doi: 10.1177/10499091221100809. | Excluded | Not A target outcome indicator |
| 15 | Salem H, Johansen C, Schmiegelow K, Winther JF, Wehner PS, Hasle H, et al. FAMily-Oriented Support (FAMOS): development and feasibility of a psychosocial intervention for families of childhood cancer survivors. Acta Oncol. 2017;56(2):367-74.Doi: 10.1080/0284186X.2016.1269194. | Excluded | Not a technology-based study |
| 16 | ChiCTR. Feasibility, acceptability and psychosocial outcomes of a smartphone-based care support program for rural families of children with cancer: a cluster randomized controlled trial. 2020. | Excluded | Just registering for the article |
| 17 | Wolters P, Abel C, Martin S, Smith K, Toledo-Tamula MA, Roderick MC, et al. Home physical activity intervention to improve cognitive late effects in children treated with radiation for brain tumors: descriptive feasibility data from a pilot randomized controlled trial (RCT). 2018;20: i168.Doi: 10.1093/neuonc/noy059. | Excluded | Conference abstract |
| 18 | NCT. Music Therapy Video Development in Improving Communication, Emotional Distress, and Recovery in Adolescents/Young Adults Undergoing Treatment for High-Risk Cancer and Their Parents. 2012. | Excluded | Just registering for the article |
| 19 | NCT. Technology-Based Psychosocial Empowerment Program for Home Care of Children With Cancer and Their Parents. 2022. | Excluded | Just registering for the article |
| 20 | NCT. Testing eSCCIP: an eHealth Psychosocial Intervention for English and Spanish Speaking Parents of Children With Cancer. 2022. | Excluded | Just registering for the article |
| 21 | ChiCTR-OPN-. The impact of an-e-health intervention on stress, quality of life, andcoping skills of parents of children with cancer: a multi-centered non-equivalent control group design. 2015. | Excluded | Just registering for the article |
| 22 | Liu SY, Sun BF, Sun YQ. Effect of family task intervention on improving family function and parental self-efficacy in children with malignant tumor. Chinese rehabilitation theory and practice. 2014(8):785-8.Doi: 10.3969/j.issn.1006-9771.2014.08.020. | Excluded | Not A target outcome indicator |
