# Supplementary material for: Impact of technology- and parent-based psychosocial interventions on family dynamics factors in children with cancer: A systematic review
Source: PLoS One. 2025 May 13;20(5):e0323483. doi: 10.1371/journal.pone.0323483 (PMC12074529; doi:10.1371/journal.pone.0323483)
Supplement: S3 File — (PDF) [file pone.0323483.s005.pdf]

Systematic review

Please select one of the options below to edit your record. Either option will create a new version of the record - the existing version will remain unchanged.

A list of fields that can be edited in an update can be found here

1. * Review title. [1 change]

Give the title of the review in English

The impact of technology and parent-based psychosocial intervention on family factors in children with cancer: a systematic review and meta analysis

2. Original language title. [1 change]

For reviews in languages other than English, give the title in the original language. This will be displayed with the English language title.

基于技术和父母的社会心理干预对癌症儿童家庭因素的影响：系统回顾和荟萃分析

3. * Anticipated or actual start date. [1 change]

Give the date the systematic review started or is expected to start.

01/08/2024

4. * Anticipated completion date. [1 change]

Give the date by which the review is expected to be completed.

30/09/2024

5. * Stage of review at time of this submission. [2 changes]

This field uses answers to initial screening questions. It cannot be edited until after registration.

Tick the boxes to show which review tasks have been started and which have been completed.

Update this field each time any amendments are made to a published record.

The review has not yet started: No

Review stage	Started	Completed
Preliminary searches	Yes	No
Piloting of the study selection process	No	No
Formal screening of search results against eligibility criteria	No	No
Data extraction	No	No
Risk of bias (quality) assessment	No	No
Data analysis	No	No

Provide any other relevant information about the stage of the review here.

6. * Named contact.

The named contact is the guarantor for the accuracy of the information in the register record. This may be any member of the review team.

Zhang Yilin

Email salutation (e.g. "Dr Smith" or "Joanne") for correspondence:
Ms Yilin

7. * Named contact email.

Give the electronic email address of the named contact.

zhangyilin1026@163.com

8. Named contact address

PLEASE NOTE this information will be published in the PROSPERO record so please do not enter private information, i.e. personal home address

Give the full institutional/organisational postal address for the named contact.

No. 172 Tongzipo Road, Yuelu District, Changsha, Hunan province

9. Named contact phone number.

Give the telephone number for the named contact, including international dialling code.

19376954996

10. * Organisational affiliation of the review.

Full title of the organisational affiliations for this review and website address if available. This field may be completed as 'None' if the review is not affiliated to any organisation.

Xiang Ya Nursing College， Central South University

Organisation web address:

11. * Review team members and their organisational affiliations.

Give the personal details and the organisational affiliations of each member of the review team. Affiliation refers to groups or organisations to which review team members belong.
NOTE: email and country now MUST be entered for each person, unless you are amending a published record.
PLEASE USE AN INSTITUTIONAL EMAIL ADDRESS IF POSSIBLE.

Zhang Yilin. Xiang Ya Nursing College， Central South University
Peng Yunyun. Xiang Ya Nursing College， Central South University
Zhang Zitong. Xiang Ya Nursing College, Central South University
Zhang Wanting. Xiang Ya Nursing College, Central South University
Professor Gu Can. Xiang Ya Nursing College， Central South University

12. * Funding sources/sponsors.

Details of the individuals, organizations, groups, companies or other legal entities who have funded or sponsored the review.

National Natural Science Foundation of China: 《The development and effectiveness of a complex family adaptation intervention strategies among families of hospitalized child with cancer》

Grant number(s)

State the funder, grant or award number and the date of award

Project approval no.: 82272924

Date:2023.1-2026.12

13. * Conflicts of interest.

List actual or perceived conflicts of interest (financial or academic).

None

14. Collaborators.

Give the name and affiliation of any individuals or organisations who are working on the review but who are not listed as review team members. **NOTE: email and country must be completed for each person, unless you are amending a published record.**

15. * Review question. [1 change]

State the review question(s) clearly and precisely. It may be appropriate to break very broad questions down into a series of related more specific questions. Questions may be framed or refined using PI(E)COS or similar where relevant.

The aim of this systematic review is to evaluate the impact of the technology and parent-based psychosocial intervention on the family factors of in children with cancer.

16. * Searches. [1 change]

State the sources that will be searched (e.g. Medline). Give the search dates, and any restrictions (e.g. language or publication date). Do NOT enter the full search strategy (it may be provided as a link or attachment below.)

Chinese literature will be searched in CNKI, Wanfang, VIP and sinomed databases.

English literature will be searched in the Cochrane Library, ProQuest, Embase, PubMed, Web of Science, Scopus databases.

Date from the establishment of the databases to the present.

17. URL to search strategy.

Upload a file with your search strategy, or an example of a search strategy for a specific database, (including the keywords) in pdf or word format. In doing so you are consenting to the file being made publicly accessible.

Or provide a URL or link to the strategy. Do NOT provide links to your search **results**.

Do not make this file publicly available until the review is complete

18. * Condition or domain being studied. [1 change]

Give a short description of the disease, condition or healthcare domain being studied in your systematic review.

Childhood cancer is the sixth leading cause of the global cancer burden. A cancer diagnosis will have serious implications for patients and their families. With advances in medical technology and treatment measures, survival rates for childhood cancer have increased significantly. As a result, this deadly disease is gradually transforming into a chronic disease, this often has adverse consequences for the psychosocial well-being of the child and his or her family. Looking at the family as a whole, there was a significant association between psychosocial stress in children with tumors and parents. Technology-based interventions have been shown to be effective in cancer-related research. So far, there is a lack of systematic evaluation of technology and parent-based psychosocial interventions for family factors in children with cancer.

19. * Participants/population. [1 change]

Specify the participants or populations being studied in the review. The preferred format includes details of both inclusion and exclusion criteria.

The parents of children who are ≤ 18 years old and diagnosed with any type of cancer.

20. * Intervention(s), exposure(s). [1 change]

Give full and clear descriptions or definitions of the interventions or the exposures to be reviewed. The preferred format includes details of both inclusion and exclusion criteria.

Technology and parent-based psychosocial intervention. The technical aspect is defined as any information or communication technology. The parental aspect is defined as including at least one of the parents.

21. * Comparator(s)/control.

Where relevant, give details of the alternatives against which the intervention/exposure will be compared (e.g. another intervention or a non-exposed control group). The preferred format includes details of both inclusion and exclusion criteria.

The control group will receive routine nursing or routine psychological nursing or health education.

22. * Types of study to be included. [1 change]

Give details of the study designs (e.g. RCT) that are eligible for inclusion in the review. The preferred format includes both inclusion and exclusion criteria. If there are no restrictions on the types of study, this should be stated.

Experimental studies (randomized controlled trials (RCTs) and quasi-experimental studies) will be included.

23. Context.

Give summary details of the setting or other relevant characteristics, which help define the inclusion or exclusion criteria.

24. * Main outcome(s). [1 change]

Give the pre-specified main (most important) outcomes of the review, including details of how the outcome is defined and measured and when these measurement are made, if these are part of the review inclusion criteria.

Family factors (such as Family communication; Family coping ability; Family function, etc.)

Measures of effect

25. * Additional outcome(s).

List the pre-specified additional outcomes of the review, with a similar level of detail to that required for main outcomes. Where there are no additional outcomes please state ‘None’ or ‘Not applicable’ as appropriate to the review

None

Measures of effect

26. * Data extraction (selection and coding).

Describe how studies will be selected for inclusion. State what data will be extracted or obtained. State how this will be done and recorded.

All search results will be imported into the reference management software EndNote X9 for data management. After all duplicate articles will be deleted, two reviewers independently screen the articles and cross-check them. Any disagreements about whether to include an article will be resolved through consultation with a third reviewer. The same two reviewers will extract the information from each article, including the year, author,

country, the design of the study, the number of samples, the characteristics of the technology-based interventions applied, the duration of the intervention, the duration of the follow-up, the important results obtained, and the theories used in the intervention.

27. * Risk of bias (quality) assessment. [1 change]

State which characteristics of the studies will be assessed and/or any formal risk of bias/quality assessment tools that will be used.

The quality assessment will be conducted using the Cochrane risk bias assessment tool for randomized controlled trials.
The quality assessment will be conducted using the Risk of Bias Assessment tool for Non-randomized Studies (RoBANS).

28. * Strategy for data synthesis. [1 change]

Describe the methods you plan to use to synthesise data. This **must not be generic text** but should be **specific to your review** and describe how the proposed approach will be applied to your data.
If meta-analysis is planned, describe the models to be used, methods to explore statistical heterogeneity, and software package to be used.

The methods appropriate for the data type among the study results will be selected for data analysis. Meta-analyses will be performed only when two or more studies had comparable treatments and outcomes. Based on available extracted data, studies will be pooled in statistical meta-analysis using RevMan V5.3 (Copenhagen: The Nordic Cochrane Centre, Cochrane). Effect sizes, expressed as odds ratios (ORs) and their 95% confidence intervals (CI) or mean differences (MD) and standardized mean differences (SMD), will be calculated for analysis. Heterogeneity across studies will be assessed by means of the I^2 index or standard χ^2. For the other outcomes where statistical pooling will be not possible, the findings will be presented in narrative form including tables to aid in data presentation where appropriate.

29. * Analysis of subgroups or subsets.

State any planned investigation of ‘subgroups’. Be clear and specific about which type of study or participant will be included in each group or covariate investigated. State the planned analytic approach.

Subgroup analysis will be performed when relevant from a clinical perspective or in the presence of significant heterogeneity.To further illustrate the subgroup analysis, forest plots will be created.

30. * Type and method of review.

Select the type of review, review method and health area from the lists below.

Type of review

Cost effectiveness	No
Diagnostic	No
Epidemiologic	No
Individual patient data (IPD) meta-analysis	No
Intervention	No
Living systematic review	No
Meta-analysis	Yes
Methodology	No
Narrative synthesis	No
Network meta-analysis	No
Pre-clinical	No

Prevention	No
Prognostic	No
Prospective meta-analysis (PMA)	No
Review of reviews	No
Service delivery	No
Synthesis of qualitative studies	No
Systematic review	Yes
Other	No

Health area of the review

Alcohol/substance misuse/abuse	No
Blood and immune system	No
Cancer	Yes
Cardiovascular	No
Care of the elderly	No
Child health	Yes
Complementary therapies	No
COVID-19	No
Crime and justice	No
Dental	No
Digestive system	No
Ear, nose and throat	No
Education	No
Endocrine and metabolic disorders	No
Eye disorders	No
General interest	No
Genetics	No
Health inequalities/health equity	No
Infections and infestations	No
International development	No

Mental health and behavioural conditions	Yes
Musculoskeletal	No
Neurological	No
Nursing	Yes
Obstetrics and gynaecology	No
Oral health	No
Palliative care	No
Perioperative care	No
Physiotherapy	No
Pregnancy and childbirth	No
Public health (including social determinants of health)	No
Rehabilitation	No
Respiratory disorders	No
Service delivery	No
Skin disorders	No
Social care	No
Surgery	No
Tropical Medicine	No
Urological	No
Wounds, injuries and accidents	No
Violence and abuse	No

31. Language. [1 change]

Select each language individually to add it to the list below, use the bin icon to remove any added in error.

English

There is not an English language summary

32. * Country.

Select the country in which the review is being carried out. For multi-national collaborations select all the countries involved.

China

33. Other registration details.

Name any other organisation where the systematic review title or protocol is registered (e.g. Campbell, or The Joanna Briggs Institute) together with any unique identification number assigned by them.
If extracted data will be stored and made available through a repository such as the Systematic Review Data Repository (SRDR), details and a link should be included here. If none, leave blank.

34. Reference and/or URL for published protocol.

If the protocol for this review is published provide details (authors, title and journal details, preferably in Vancouver format)

No I do not make this file publicly available until the review is complete

35. Dissemination plans.

Do you intend to publish the review on completion?

No

36. Keywords.

Give words or phrases that best describe the review. Separate keywords with a semicolon or new line. Keywords help PROSPERO users find your review (keywords do not appear in the public record but are included in searches). Be as specific and precise as possible. Avoid acronyms and abbreviations unless these are in wide use.

37. Details of any existing review of the same topic by the same authors.

If you are registering an update of an existing review give details of the earlier versions and include a full bibliographic reference, if available.

38. * Current review status. [1 change]

Update review status when the review is completed and when it is published.
New registrations must be ongoing so this field is not editable for initial submission.

Review_Ongoing

39. Any additional information.

Provide any other information relevant to the registration of this review.

40. Details of final report/publication(s) or preprints if available.

Leave empty until publication details are available OR you have a link to a preprint (NOTE: this field is not editable for initial submission).
List authors, title and journal details preferably in Vancouver format.
